# A Win–Win Combination to Inhibit Persistent Organic Pollutant Formation via the Co-Incineration of Polyvinyl Chloride E-Waste and Sewage Sludge

**DOI:** 10.3390/polym13050835

**Published:** 2021-03-09

**Authors:** Gerard Gandon-Ros, Samuel S. Nuñez, Nuria Ortuño, Ignacio Aracil, María Francisca Gómez-Rico, Juan A. Conesa

**Affiliations:** 1 Institute of Chemical Process Engineering, University of Alicante, P.O. Box 99, E-03080 Alicante, Spain; gerard.gandon@ua.es (G.G.-R.); samu1228@gmail.com (S.S.N.); nuria.ortuno@ua.es (N.O.); nacho.aracil@ua.es (I.A.); paqui.gomez@ua.es (M.F.G.-R.); 2 Department of Chemical Engineering, University of Alicante, P.O. Box 99, E-03080 Alicante, Spain

**Keywords:** Inhibition, co-combustion, co-incineration, PVC, sewage sludge, dioxin, organic compounds

## Abstract

Persistent organic pollutant inhibition in the combustion process of polyvinyl chloride (PVC) by prior addition of an inhibitor is currently being studied, reducing the emission of pollutants, and thus reducing the large amount of waste PVC destined for landfill. In this work, the use of sewage sludge (SS) as an alternative to chemical inhibitors to improve the quality emissions of the incineration of polyvinyl chloride waste (PVC e-waste) was studied and optimized. Different combustion runs were carried out at 850 °C in a laboratory tubular reactor, varying both the molar ratio R_i_ (0.25, 0.50, 0.75) between inhibitors (N + S) and chlorine (Cl) and the oxygen ratio λ (0.15, 0.50) between actual oxygen and stoichiometric oxygen. The emissions of several semivolatile compounds families such as polycyclic aromatic hydrocarbons (PAHs), polychlorobenzenes (ClBzs), and polychlorophenols (ClPhs), with special interest in the emissions of the most toxic compounds, i.e., polychlorinated dibenzo-*p*-dioxins and polychlorinated dibenzofurans (PCDD/Fs) and dioxin-like polychlorinated biphenyls (dl-PCBs), were analyzed. A notable decrease in PCDD/F and dl-PCB formation was achieved in most of the experiments, especially for those runs performed under an oxygen-rich atmosphere (λ = 0.50), where the addition of sludge was beneficial with inhibition ratios Ri ≥ 0.25. An inhibition ratio of 0.75 showed the best results with almost a 100% reduction in PCDD/F formation and a 95% reduction in dl-PCB formation.

## 1. Introduction

Polyvinyl chloride (PVC) e-waste is a highly chlorinated thermoplastic whose generation has significantly increased in the last years and its disposal involves nowadays a substantial environmental uncertainty [1]. In fact, PVC incineration is considered as a potential source of emission of toxic and harmful pollutants such as chlorinated organic compounds [2]. During the process of waste incineration, an involuntarily and considerable amount of toxic polychlorinated dioxins and furans (PCDD/Fs), polychlorinated biphenyls (PCBs), as well as polycyclic aromatic hydrocarbons (PAHs), among others, can be formed under certain operating conditions. The emission of PCDD/Fs and PCBs in the primary combustion chamber is proved to be higher when incinerating chlorine-composed wastes [3].

**Figure 1:**
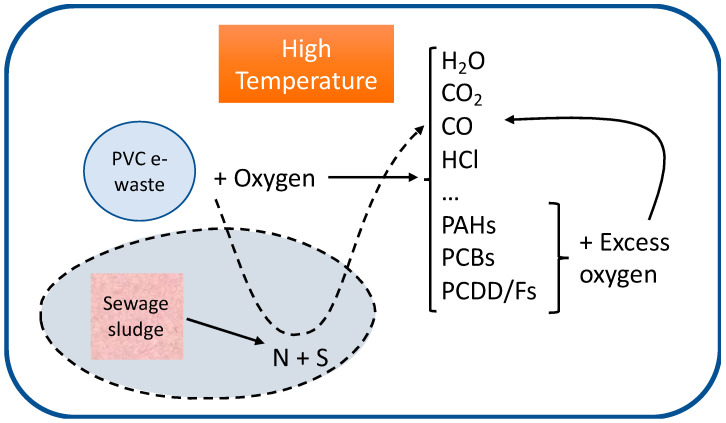
Scheme of the processes taking place in the decomposition of polyvinyl chloride (PVC) at high temperature in the presence of oxygen and nitrogen + sulfur coming from sewage sludges.

A simple scheme of the process occurring during thermal decomposition of wastes is shown in Figure 1. In conditions of low presence of oxygen, or bad mixing conditions, the decomposition of a waste, particularly when the chlorine content is high, can produce a variety of pollutants. The maximum formation of the 16 priority PAHs listed by the US Environmental Protection Agency (US EPA) occurs under pyrolytic conditions (no oxygen) at high temperatures (850 °C), as expected since it is known that pyrolytic reactions are the primary source of PAH formation [4,5]. When oxygen is fed at higher amounts, usually pollutant emission decreases, if temperature is high enough. For example, PAHs clearly show a minor emission as the oxygen ratio increases when working at high temperature, and this trend has been observed during the decomposition of many wastes studied. This indicates that PAHs are pyrolytic products that are easily eliminated in oxygen–rich environments at high temperature [5].

At present, many studies have been performed to reduce the emission of PCDD/Fs formed from the thermal treatment of waste with different methods, such as photocatalytic decomposition, catalytic oxidation, and through ozone application [6,7,8]. However, to prevent the formation of these contaminants, chemical inhibition is the most promising method. Inhibitor compounds must be efficient, non-toxic and inexpensive. For these reasons, in recent years studies have been carried out mainly with 4 types of inhibitors: Metallic oxides, nitrogen compounds, sulfur compounds, and nitrogen and sulfur compounds, the last type being the ones that provide the best results [9,10].

Most studies focus on the use of specific nitrogen- and sulfur-based compounds (thiourea, sulfamic acid, sulfate, and ammonium thiosulfate), obtaining good results of inhibition in PCDD/F formation and even reducing also NOx emissions [11,12,13]. Co-combustion of biomass with waste from the paper industry, with a high chlorine content, has already been proven to reduce PCDD/F and PCB emissions, using ammonium sulfate as nitrogen and sulfur inhibitor [14].

In addition, the possibility of using waste rich in nitrogen and/or sulfur as a source of inhibitory compounds is getting more and more relevance because of the reduced pollutant emission factor, besides reducing waste accumulation in landfills. In this way, the effect of adding sludge from urban wastewater treatment plants (WWTPs), which have a high content of nitrogen compounds, has been specially studied in three different ways: By direct mix of the waste [15], by using the gas coming from drying sludge [16,17] and by using the pyrolysis gas of sludge together with certain compounds [18].

Sewage sludge (SS) is a semi-solid waste that is generated in urban WWTPs as a consequence of water treatment processes. The use of SS is normally linked to obtaining compost for its use in agriculture as main destination. However, the large amounts generated and the fact that not all the SS can be devoted to its use as compost due to its pollutant load, makes its use as an alternative fuel be the main option. Moreover, it is worth mentioning that during the compost production process from this waste, PCDD/Fs could be generated in certain situations [19,20,21]. Although there are already some studies in relation to the possible use of sewage sludge as an inhibitor of PCDD/F formation in thermal treatments of other wastes [15], the ideal conditions for its use remain uncertain, especially for a full e-waste.

Therefore, given the nitrogen and sulfur content of SS and high chlorine content of PVC e-waste, this work aims to study the potential of combining both by direct mixture in a co-incineration process to reduce the emission of PCDD/Fs, PCBs and other organic micropollutants which are incomplete combustion products. The study results could be applied in thermal treatment units such as incinerators, blast furnaces and furnaces of cement and ceramics production. In this way, it could help in reducing the amount of pollutants produced, too.

In addition, this work is in line with the sustainable development goals (SDGs) 3, 9 and 11, stablished in the 2030 agenda for sustainable development by the United Nations, which aim for a pollution-free planet. Environmental degradation is responsible for nearly one in four deaths (12.6 million people a year), as well as a variety of health problems and widespread destruction of vital ecosystems, and in particular air pollution, which has been estimated to cause about 6.5 million deaths per year [22].

## 2. Materials and Methods

### 2.1. PVC E-Waste

Electrical wires used in this study were supplied by General Cable, Co (Barcelona, Spain). These wires were composed by a PVC cover and a cross-linked polyethylene (PE) insulator with copper as a conductor, already used in previous works [23,24,25,26,27].

Nevertheless, in the present work only the plastic fraction formed by the cover and the insulation material were used, consisting of e-waste of PVC (82.8%) with impurities of PE (17.2%). This plastic fraction was manually separated from the metallic fraction and then crushed using a cutting mill (Retsch SM 200, Haan, Germany) until obtaining a particle size of less than 1 mm, small enough to guarantee the homogeneity of the samples in the runs.

This e-waste was previously characterized employing an elemental microanalyzer (Thermo Finnigan Flash 1112 Series) to furnish total content (wt%) of carbon, hydrogen, nitrogen and sulfur, and an X-ray fluorescence spectrometer (Philips Magix Pro PW2400, Eindhove, Netherlands) to provide the rest of elements [28]. Table 1 shows the elemental analysis and composition of the PVC e-waste. A high amount of calcium is found in the sample, probably due to the presence of calcium carbonate, usually employed in wire/PVC formulations to reduce the price of the compound while improving electrical or physical properties. In the present case, a high amount of calcium is measured, but the methodology used for this analysis (X-ray Fluorescence) is a semi-quantitative technique.

### 2.2. Sewage Sludge (SS)

SS were provided by Cemex España SA (Alicante, Spain) and prior to their use, SS were dried in an oven at 105 °C for 24 h and reduced to dust by a mixer. The ash content was measured following the UNE-EN-14775:2009 [29] at 550 °C, and was 20.56 wt% (average of three duplicates). The concentration of bromine and chlorine were measured using the US EPA Methods 5050 [30] and 9056 A [31] by oxygen combustion bomb-ion (Dionex DX-500, Sunnyvale, CA, USA), obtaining from an average of two duplicates a negligible amount of 0.008 wt% bromine and 0.363 wt% chlorine. In addition, the net calorific value (NCV) was measured from three duplicates, obtaining an average value of 19.2 KJ/g. The results of the elemental analysis and X-ray fluorescence analysis carried out for this sewage sludge are shown in Table 2.

### 2.3. Experimental System and Operating Conditions

The different experiments performed to determine the different compounds produced during the combustion of PVC and sludge were carried out in a horizontal reactor with a combined displacer-furnace system.

The reactor has been used in previous works [23,26] and consists of a quartz tube with an external diameter of 10 mm, a wall thickness of 1 mm and a length of 1 m, where the sample was introduced ensuring a homogeneous distribution. For the introduction of the samples into the reactor, quartz boats with an external diameter of 7 mm and a length of 70 mm were used, arranged in series with no space between them. This system allowed the decomposition of sample quantities up to approximately 1 g when two boats were employed.

To introduce the sample into the combustion zone, a linear actuator (IAI America Inc., Shizuoka, Japan) with a movement capacity up to 500 mm and speeds between 0.1 and 800 mm/s was used. The reproducibility of the actuator (±0.02 mm/s) ensured a homogeneous feed flow. The actuator was coupled to a controller that allows the creation of different movement programs, by defining the initial and final points, as well as the speeds associated with the trajectories between programmed points. This equipment allows the sample to move in parallel towards the combustion zone in the same direction as the air flow, with a maximum flow rate of 500 mL/min.

The mixtures of both wastes were made following a “molar inhibition ratio” that relates the amount of nitrogen and sulfur contained in the sludge, with the amount of chlorine contained in PVC e-waste. The molar inhibition ratio (R_i_) was defined according to the Equation (1):

(1) $$R_{i}=\frac{\left(S+N\right)}{\mathrm{Cl}}$$

where S, N and Cl were the molar fraction of sulfur, nitrogen, and chlorine, respectively; values of R_i_ = 0.25, R_i_ = 0.50, and R_i_ = 0.75 were considered.

In the same way, the effect of combustion atmosphere in pollutant formation was examined by varying the oxygen ratio (λ). Oxygen ratio (λ) was calculated based on the synthetic air supplied to the experiment with the Equation (2):

(2) $$\lambda =\frac{{\mathrm{Oxygen}}^{\mathrm{actual}}}{{\mathrm{Oxygen}}^{\mathrm{stoichiometric}}}=\frac{{\mathrm{Air}}^{\mathrm{actual}}}{{\mathrm{Air}}^{\mathrm{stoichiometric}}}$$

where Air^actual^ and Air^stoichiometric^ were, respectively, the actual and the stoichiometric air flow rate necessary for a complete combustion [32,33].

In this way, the oxygen ratio would be λ = 1 for a combustion process where the oxygen present is the stoichiometric one and λ = 0 for a reaction with total absence of oxygen. For each experiment, the oxygen ratio was adjusted by changing the speed of the samples entering the combustion chamber with values between 6 ×·10^−4^ and 1.6·× 10^−3^ m/s, as well as the air flow rate with values between 400 and 589 mL/min. Values of λ = 0.15, and λ = 0.50 were considered.

Once synthetic air flow rate (measured at 1 atm and 20 °C) and horizontal furnace temperature were attained and constant (850 °C in every run performed), the boats were introduced in parallel with air flow at constant speed with the appropriate sample load. Before each experiment, a control run with no sample loading was carried out using the same experimental conditions (blank).

The whole study was performed with a total of eleven samples, including blank experiments. Apart from PVC e-waste and sewage sludge, the samples consisted of 3 mixtures of sewage sludge and PVC e-waste in different proportions of (S + N)/Cl (R_i_ = 0.25, R_i_ = 0.50 and R_i_ = 0.75). For every mixture, experiments were carried out under two atmospheres with different amounts of oxygen (λ = 0.15 and 0.50).

A total of 11 experiments were run following specific conditions as shown in Table 3:

Since the reproducibility of this type of experiments has already been assessed in our laboratory with a relative standard deviation of only 5% [34], a single experiment was carried out for each experimental condition. All the semivolatile compounds to be studied (PAHs, ClBzs, ClPhs, PCDD/Fs, and PCBs) were adsorbed on a fixed bed of Amberlite^®^ XAD-2 polyaromatic resin located at the outlet of the reactor. The resin was previously washed by means of three consecutive solid–liquid extractions with methanol, dichloromethane and toluene in the ASE^®^ 100 extractor and was dried up at room temperature.

All compounds were analyzed from a single resin for each sample, sharing the pre-treatment process, but not their analysis, identification and quantification.

The internal standards used to quantify all the semivolatile compounds analyzed were added to the resin before the extraction process [35]. Internal standard MIX26 for PAHs analysis was purchased from Dr. Ehrenstorfer-Schäfers and internal standards MCBS, MCPS, EPA-1613LCS, and WP-LCS for ClBzs, ClPhs, PCDD/Fs, and PCBs analysis, respectively, were supplied by Wellington Laboratories.

The extraction process of the resin samples was performed in an ASE^®^ 100 extractor (Accelerated Solvent Extractor). For the extraction of the samples, pure toluene was used as solvent, since the extraction of all the compounds was done together and toluene must be used to extract the heaviest compounds. Once the extract was obtained, it was separated into two fractions. A first fraction with 30% of the total weight of the extract was used for the analysis of PAHs, ClBzs, and ClPhs, and a second fraction with the remaining 70% of the extract was used for the analysis of PCDD/Fs and PCBs. Both extract fractions were concentrated on a Büchi model R210/V rotary evaporator until a final volume of around 1 mL.

Fraction 1 samples were transferred to a chromatography vial to proceed to the concentration with a N_2_ steam in a multiple Pasvial evaporator until a volume of approximately 1.5 mL was reached prior to the analysis by high-performance gas chromatography (Agilent 6890N) with mass spectrometry (Agilent 5973N) (HRGC-MS) as explained elsewhere [26]. Three µL of anthracene-d_10_ with a concentration of 2000 µg/mL in dichloromethane (AccuStandard Inc., New Haven, CT, USA) was added as a recovery standard. The addition of a known quantity of this standard allows to calculate the recovery percentage of the internal standards throughout the process.

Fraction 2 samples, for the analysis of PCDD/Fs and PCBs, were purified using a Power-Prep^TM^ automatic cleaning equipment, from the company FMS, which is an automated fluid management system, capable of automatically purifying extracts from samples of different nature for the subsequent analysis of compounds toxic at trace levels. This system uses three different disposable columns supplied by the manufacturer: An acid-base multilayer silica column, an alumina column, and an activated carbon column. PAHs, phenols, acids, and esters, as well as oils and lipids, are retained on the silica column. The basic alumina column allows to separate PCDD/Fs from other organic compounds and also retains lipids and phenols, separating the PCB fraction (both non-ortho and mono-ortho). Finally, the activated carbon column allows the PCDD/Fs to be isolated from other organic compounds, as they are retained at the top of the column due to their planar configuration, being subsequently eluted in reverse flow.

After purification, two fractions were obtained: One containing the PCDD/Fs in toluene and the other containing the PCBs in a 50% mixture of dichloromethane/hexane (*v*/*v*). Both fractions were concentrated in the rotary evaporator to an approximate volume of 1 mL. Next, 50 µL of nonane was added and the sample concentration was completed with the aid of a gentle stream of N_2_ to a final volume of about 50 µL. Just before the analysis, the ^13^C-isotopically labeled recovery standards were added to the different fractions (PCDD/Fs and PCBs), which allowed to calculate the percentage of recovery or loss of the internal standards throughout the extraction, purification and concentration process. Specifically, 10 μL of the EPA-1613ISS solution (Wellington Laboratories, Guelph, Canada) for PCDD/Fs and 10 μL of the WP-ISS solution diluted to 200 ppm (Wellington Laboratories) for PCBs were employed.

The analysis of PCDD/Fs and dioxin-like PCBs was carried out by gas chromatography (Agilent 7890B) coupled to triple quadrupole mass spectrometry (Agilent 7010B) (GC-MS/QQQ). The system is also equipped with an Agilent 7693A automatic injector. Table S1 lists the instrument conditions. Both calibration and tuning of the instrument were repeated periodically using the EI high sensitivity autotune mode, and the instrument performance was verified at least on a weekly basis.

The methods use Multiple Reaction Monitoring (MRM) mode for data acquisition. For each target, two specific precursor ions as well as two corresponding product ions and collision energies were selected: One MRM transition for quantitation and one for qualification. Tables S2 and S3 give a full list of MRM transitions and collision energies for PCDD/Fs and PCBs, respectively. Quantitation was performed with the quantitative transition only, while the qualitative transition was used to verify the ion ratio between the two transitions.

The quantification of PCDD/Fs and PCBs was carried out by isotope dilution methodology, which uses the relative response factor for each congener with respect to the corresponding labeled standard, following the requirements of the U.S. EPA method 1613 [36] for dioxins and method 1668C [37] for dl-PCBs. Data analysis was performed with Agilent MassHunter Quantitative Analysis Software (version B.09.00).

## 3. Results and Discussion

### 3.1. PAHs

In order to check that the results obtained were reliable, the recovery of the deuterated standards for each sample was verified. The results obtained for the recoveries of the deuterated standards in all the experiments performed at λ = 0.15 were around 100%, with the exception of the recovery value of chrysene-d_12_ for an R_i_ of 0.75, which was slightly higher. For λ = 0.50, the recoveries were between 40% and 100%.

**Figure 2:**
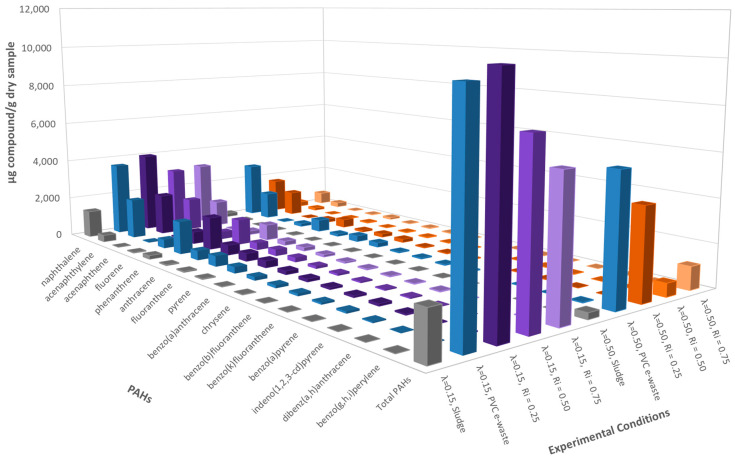
Emissions of polycyclic aromatic hydrocarbons (PAHs) (ppm) for every experiment run at 850 °C and both oxygen ratios, λ = 0.15 and λ = 0.50.

The results obtained for the 16 priority PAHs were determined using the isotope dilution technique and are presented in Figure 2.

#### 3.1.1. Emissions of PAHs

Figure 2 shows the emission of the different PAHs and their total yield, during the decomposition of the pure samples and the different mixtures prepared. The lowest concentrations of these pollutants were obtained during the combustion of sludge, whereas higher concentrations were expected from PVC decomposition.

The decomposition with a λ = 0.15 presented much higher concentrations of PAHs than runs at λ = 0.50, as expected due to a less efficient combustion [26]. Values of PAH emission were intermediate for R_i_ = 0.50 and R_i_ = 0.75, as could be expected even in the case of no inhibition action for the mixture of both residues. However, for R_i_ = 0.25 the total concentration of PAHs was even slightly above the values reached for the pure PVC e-waste sample.

For λ = 0.50, the highest concentrations of PAHs were obtained for PVC e-waste and the lowest concentrations were obtained for sludge. This time, lower intermediate concentrations of PAHs were obtained for the mixtures, even when an increase in the inhibition ratio was not directly correlated with a decrease in the concentration of PAHs, as observed for λ = 0.15. In this case, the lowest concentration of PAHs was obtained for a R_i_ = 0.50; this is due to the presence of sufficient oxygen in the atmosphere to remove a large part of the hydrocarbons formed in the pyrolytic stages.

PVC e-waste contained around 30 times more chlorine than sludge (see Table 1 and Table 2), so a higher inhibition ratio implies a lower total amount of chlorine in the samples. In general, a clear profile of decreasing concentrations was observed, directly related to the amount of PVC e-waste contained in the sample and obtaining lower amounts of PAHs as the proportion of this waste decreased.

#### 3.1.2. Inhibition of PAH Formation

**Figure 3:**
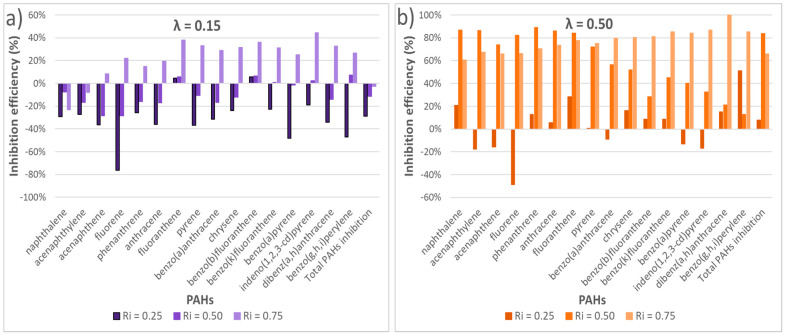
Inhibition efficiency (%) in PAH formation for every experiment run at 850 °C and both oxygen ratios: (**a**) λ = 0.15, and (**b**) λ = 0.50.

In order to be more accurate and to determine if the sludge acted effectively as an inhibitor in their formation, the concentration of PAHs that should have been obtained (expected) in the samples PVC/sludge simply considering their independent contributions was calculated based on the sludge and PVC e-waste fractions of each mixture. The results of the comparisons of the expected theoretical concentrations and those actually obtained are presented in Figure 3, which allow us to detect a reduction in the formation of pollutants based on the different inhibition ratios used.

The percentages of reduction in the formation of PAHs were calculated for the different inhibition ratios and for both oxygen ratios λ = 0.15 (Figure 3a) and λ = 0.50 (Figure 3b).

As can be seen in Figure 3a, for an oxygen ratio of 0.15 and an inhibition ratio of 0.25 there was an increase in the concentration of the compounds analyzed, which means that under these conditions, mixing was disadvantageous to the reduction of contaminants since these concentrations increased in most of the compounds with respect to those expected as a result of the sum of both residues of the mixture. Acetylene was among the main organic light gases detected. In addition, as explained for PCDD/Fs emissions later in the manuscript, this increase in the concentration of the compounds analyzed could be due to the presence of catalysts in the composition of sludge. In this case, sludge was composed by around 1% of magnesium, among other metals in much less proportion, such as copper (0.09%). According to [38], the formation of PAHs could arise from acetylene interacting with olivine Mg^2+^ ions.

For R_i_ = 0.50, the concentration of undesirable compounds also increased in most of the cases and in the total amount of PAHs emitted. However, this increase in PAHs formation went from being around 30% for R_i_ = 0.25 to 15% for R_i_ = 0.50. Therefore, there was a general inhibition, compared to the experiment carried out with an R_i_ = 0.25.

For an inhibition ratio of 0.75, a significant reduction in the concentration of PAHs was obtained with respect to the expected theoretical amount, producing a significant inhibition of between 20 and 40%, with the only exceptions of naphthalene and acenaphthylene. Based on the experiments performed, it could be concluded that in order to obtain a significant reduction in some of the PAHs formed when working in an atmosphere very poor in oxygen (λ = 0.15), the best inhibition ratio was 0.75, but it would be recommended not to co-incinerate both wastes as naphthalene, acenaphthylene and phenanthrene are precisely the major compounds emitted and are the least inhibited.

The reduction in the concentration of pollutants at a low inhibition ratio begins to be noticeable when working with higher oxygen ratios. It can be seen in Figure 3 that for the same inhibition ratio, the emissions of PAHs are considerably higher for λ = 0.15 than for λ = 0.50.

As the inhibition ratio increased, the concentration of PAHs present in the sample decreased notably, some of the compounds being reduced by more than 80% (Figure 3b).

For an R_i_ of 0.75, a high percentage of inhibition between 60 and 80% was observed for every compound, reaching 100% for dibenz(a,h)anthracene. For R_i_ = 0.50 a greater reduction was observed in the lightest PAHs, which were in turn the most abundant. Therefore, although for R_i_ = 0.75 a greater reduction was obtained on average in all PAHs, at a global level the results obtained for R_i_ = 0.50 were better.

As a conclusion, λ = 0.50 was always better to reduce the emission of PAHs, so in the presence of more oxygen, the formation of PAHs is lower, being R_i_ = 0.50 and R_i_ = 0.25 the mixtures that showed the best and worst results, respectively.

### 3.2. ClBzs and ClPhs

The analysis of the results obtained from the combustion of the samples for the formation of these compounds is presented jointly, since the analysis methods were similar, and both were measured in the mass spectrometer in SIR mode.

The results obtained for the recoveries of the ^13^C-labeled standards for all the experiment runs in ClBz analysis were around 100%, except for the first two ^13^C-labeled standards, whose recoveries were around 20%. As these two were more volatile, their losses were more likely in the evaporation phase.

For the ClPhs, the recoveries of the ^13^C-labeled standards were between 40 and 80% for all the runs, with the exception of the last standard whose recovery was around 15%. This could be due to the fact that it is a heavier and more thermolabile compound, which can degrade further on the chromatographic column during GC-MS analysis.

#### 3.2.1. Emissions of ClBzs

**Figure 4:**
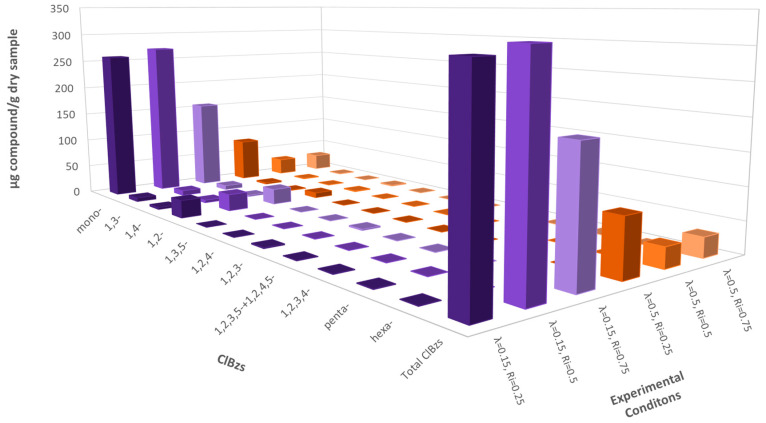
Emissions of ClBzs (ppm) for every experiment run at 850 °C and both oxygen ratios, λ = 0.15 and λ = 0.50.

Figure 4 shows the results obtained for the ClBzs at λ = 0.15 and λ = 0.50 for the different mixtures (R_i_). The results at λ = 0.15 do not show a great difference when varying inhibition ratios. For those compounds appearing with the highest concentrations, it was observed that the best inhibition ratio was 0.75. Clearly, monochlorobenzene was the isomer obtained with the highest yields, as usual in thermal treatments.

The results of ClBzs emissions at λ = 0.50 barely showed any difference between the inhibition ratios of 0.50 and 0.75. However, the worst results were clearly obtained for the inhibition ratio of 0.25, most probably due to the fact that the samples with a lower R_i_ were those which contained more chlorine and, therefore, facilitate the formation of these compounds.

The best oxygen ratio for every inhibition ratio was undoubtedly λ = 0.50, where the concentration of the compounds was approximately 6 times lower for all cases, facilitated by a predominance of decomposition mechanisms due to the higher amount of oxygen present during the thermal treatment.

#### 3.2.2. Emissions of ClPhs

**Figure 5:**
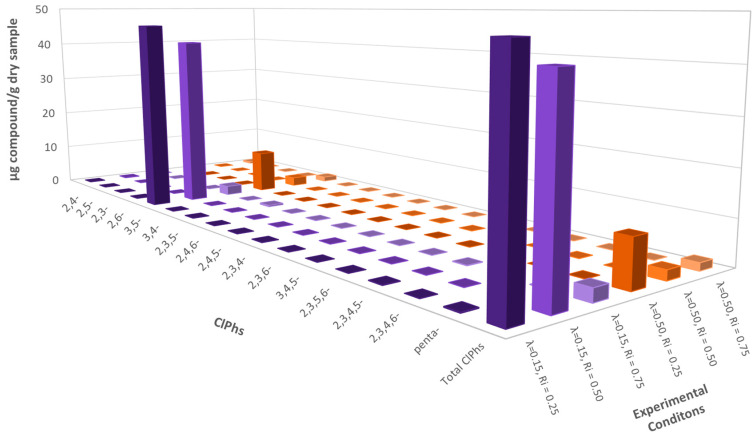
Emissions of polychlorophenols (ClPhs) (ppm) for every experiment run at 850 °C and both oxygen ratios λ = 0.15 and λ = 0.50.

In Figure 5, the results obtained for ClPhs are presented. The concentration of all the compounds was close to the detection limit of the method (0.01 ppm), except for 3,5-dichlorophenol, in which case the lowest concentrations were obtained for an inhibition ratio of 0.75 for both oxygen ratios (λ = 0.15 and λ = 0.50).

In the same way as for ClBzs, the best oxygen ratio for the mixtures with inhibition ratios 0.25 and 0.50 was λ = 0.50.

### 3.3. PCDD/Fs

The results obtained for the recoveries of the ^13^C-labeled dioxin standards in all the experiments performed at both oxygen ratios were close to 100%, except for some runs at λ = 0.15 which were between 80% and 90%. However, all values were well within the ranges allowed by the US EPA method 1613.

#### 3.3.1. Emissions of PCDD/Fs

**Figure 6:**
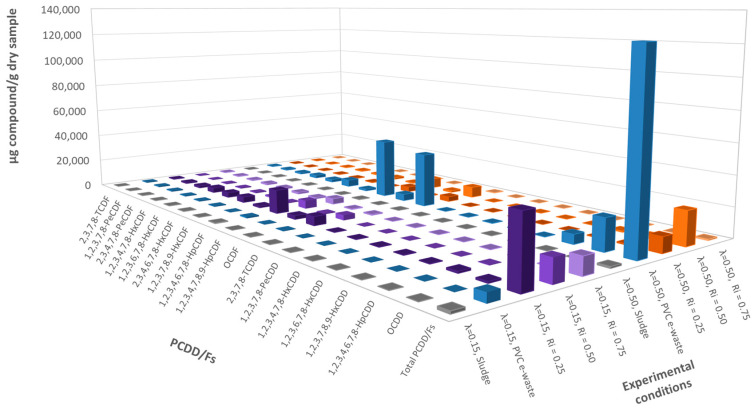
Emissions of PC polychlorinated dioxins and furans (PCDD/Fs) (ppm) for every experiment run at 850 °C and both oxygen ratios λ = 0.15 and λ = 0.50.

The results obtained at different inhibition ratios and oxygen ratios are presented in Figure 6. The results obtained for PCDD/Fs at λ = 0.15 did not correspond to the expected results. In fact, intermediate values were expected between those of sludge and PVC e-waste, due to the lower chlorine content and the inhibitory effect of N and S. As it can be seen in Figure 6 for λ = 0.15, the values for the tetra- and pentachlorinated congeners showed the highest concentrations in the PVC e-waste sample (although in this case, the total emitted amount is very low) and in the mixture R_i_ = 0.25. However, for the hexa-, hepta- and octachlorinated congeners, the mixture R_i_ = 0.25 presented higher dioxin emissions. The explanation of this phenomenon could be related to the composition of the sludge, which is shown in Table 2. Sludge was composed by around 6% of iron, among other metals in much less proportion, such as copper (0.09%). Previous studies showed that the presence of these metals catalyzes the formation reaction of PCDDs and PCDFs [23]. According to Fujimori et al. [39], some of the catalysts are FeCl_3_·6H_2_O, FeCl_2_·4H_2_O, Fe_2_O_3_, CuCl_2_·2H_2_O, and CuCl_2_·2H_2_O, which could already exist in the sample previously or be produced during the combustion process.

The lowest emissions in the case of the mixtures for λ = 0.15 were obtained for an Ri of 0.75 and the highest emissions for Ri = 0.25. For an inhibition ratio of 0.25, the obtained concentrations were much higher than for the other ratios, being this sample the one that contained the most amount of chlorine of all the mixtures tested, in addition to having small amounts of metals (iron and copper) that seemed to have catalyzed the formation reactions of PCDD/Fs.

For λ = 0.50 (greater presence of oxygen), the effect of the presence of dioxin formation catalysts in the mixtures was less evident, and what happened at λ = 0.15 when the formation of PCDD/Fs in the mixtures exceeded that of the PVC sample was not observed. Here it is important to remark the high PCDD/Fs emissions for the PVC e-waste alone at λ = 0.50 compared to the rest of the experiments.

Observing the results for different inhibition ratios at λ = 0.50, the lowest emissions were obtained for an inhibition ratio of 0.75, as in the case of λ = 0.15, being in general lower the levels of PCDD/Fs that were emitted.

It can be observed that when working with small inhibition ratios (R_i_ = 0.25) or with high inhibition ratios (R_i_ = 0.75), λ = 0.50 was always better to reduce the concentration of PCDD/Fs. However, for intermediate inhibition ratios around 0.50, the emissions obtained were similar for both oxygen ratios.

#### 3.3.2. Inhibition of PCDD/F Formation

**Figure 7:**
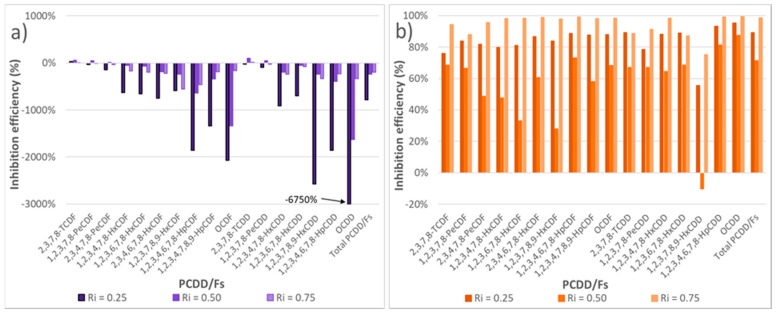
Inhibition efficiency (%) in PCDD/Fs formation for every experiment run at 850 °C and both oxygen ratios: (**a**) λ = 0.15, and (**b**) λ = 0.50.

In order to assess the effect of the different mixtures, the percentages of reduction in the formation of PCDD/Fs with respect to the expected theoretical yields were calculated for the different inhibition ratios, and for both oxygen ratios λ = 0.15 (Figure 7a) and λ = 0.50 (Figure 7b).

It was observed that in an atmosphere very poor in oxygen (λ = 0.15), the addition of sludge hindered the process, causing an increase in the concentration of PCDD/Fs that were emitted, probably due to the catalytic effect of metals discussed in the previous section.

For λ = 0.50, very promising results were obtained for every inhibition ratio studied, being the optimum ratio R_i_ = 0.75 where practically all the congeners reached a 100% reduction. The worst inhibition ratio was 0.50, reaching reductions between 30 and 80%. In this way, the global inhibition efficiencies achieved were 89.2%, 71.4%, and 98.8% for the inhibition ratios 0.25, 0.50 and 0.75, respectively.

As a conclusion, R_i_ = 0.75 was always better to minimize the emission of PCDD/Fs, so in the presence of more sludge, the formation of PCDD/Fs is much lower for both oxygen atmospheres, being λ = 0.50 the oxygen ratio that clearly showed the best results in terms of the inhibition efficiency.

### 3.4. PCBs

The results obtained for the recoveries of the ^13^C-labeled PCB standards for all the experiments performed with λ = 0.15 and λ = 0.50 were around 100%, with small variations, but within the limits allowed by the US EPA 1668 method for PCBs.

#### 3.4.1. Emissions of PCBs

**Figure 8:**
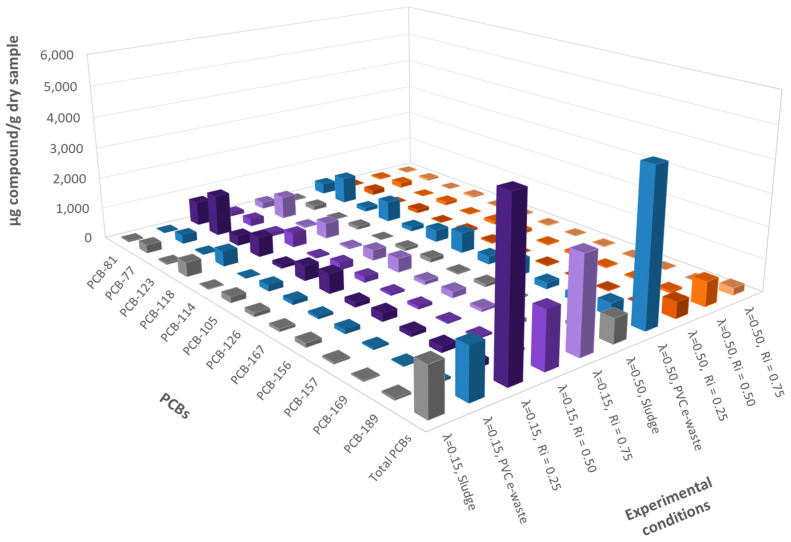
Emissions of dl-PCBs (ppm) for every experiment run at 850 °C and both oxygen ratios λ = 0.15 and λ = 0.50.

The results obtained for PCBs emissions are shown at different inhibition ratios and oxygen ratios, including pure materials, in Figure 8.

The emissions obtained from PCBs for λ = 0.15 and an inhibition ratio of 0.50 showed relatively small differences compared to sludge and PVC e-waste. However, the emissions were even higher for mixtures with R_i_ = 0.25 and R_i_ = 0.75, especially for an R_i_ = 0.25. Despite slight variations, it can be concluded that the lowest concentrations were obtained for an inhibition ratio of 0.50, which means that intermediate sludge-PVC e-waste mixtures were those with the greatest reduction in PCBs emissions. When working in an atmosphere very poor in oxygen, adding high amounts of sludge to the mix to try to inhibit the formation of PCBs should be avoided, because the opposite effect could occur. Consequently, it is crucial to determine the optimal mixture to ensure a reduction in the formation of PCBs, knowing that it lies between an inhibition ratio of 0.25 and 0.75.

When considering the concentrations obtained for PCB emissions for λ = 0.50, different trends were observed from those obtained for λ = 0.15. As can be seen in Figure 8, in this case the highest concentrations were obtained for PVC e-waste, while the concentrations of PCBs in the sludge and in the sludge-PVC e-waste mixtures were in the same order, obtaining higher emissions with the mixture R_i_ = 0.50 for the less chlorinated congeners. The increase in the emission of PCBs for λ = 0.50 with respect to λ = 0.15 was only noticeable for PVC e-waste emissions.

For λ = 0.50 the best inhibition results were obtained with R_i_ = 0.75. It can be concluded that when working at higher oxygen ratios, the use of inhibition ratios higher than 0.50 would be beneficial.

Increasing the value of the oxygen ratio from 0.15 to 0.50 showed a variation in the results at different inhibition ratios. In this later case, it was observed that the worst inhibition ratio was 0.50, when for the case of λ = 0.15, it was the relation for which the best results were obtained.

For all mixtures, an oxygen ratio λ = 0.50 was always better to reduce PCB emissions.

#### 3.4.2. Inhibition of PCB Formation

**Figure 9:**
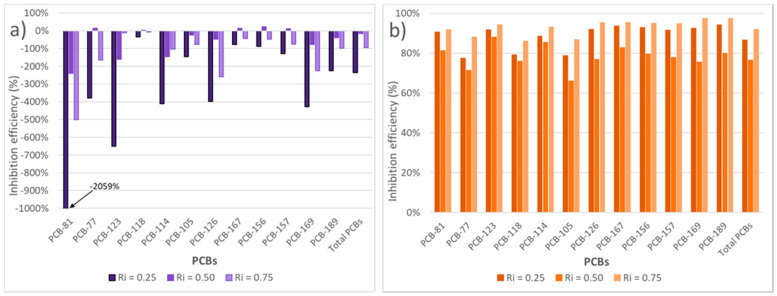
Inhibition efficiency (%) in dl-PCBs formation for every experiment run at 850 °C and both oxygen ratios: (**a**) λ = 0.15, and (**b**) λ = 0.50.

Once the best inhibition ratios that should be used at each oxygen ratio were determined, the percentages of reduction of PCBs were calculated for the different inhibition ratios and oxygen ratios, as shown in Figure 9.

For λ = 0.15, the results obtained in the study of the inhibition capacity of the sludge addition were unfavorable for all the mixtures tested, producing basically an increase of the emission of PCBs in all congeners and being the inhibition ratios of 0.25 and 0.50 the ones that provided the worst and best results, with an increase of 236.5% and 15.4% in total emissions of PCBs, respectively.

For λ = 0.50, the results obtained were really favorable in all cases, obtaining a decrease in the concentration of PCBs for all mixtures and being the inhibition ratio of 0.75 the one that provided the best results reaching a global inhibition efficiency of 91.9%.

It can be concluded that for all mixtures, an oxygen ratio λ = 0.50 was always better and even imperative to reduce PCB emissions.

## 4. Conclusions

In this work, samples of PVC e-waste, sludge, and mixtures with different inhibition ratios (R_i_), which refers to the proportion between the sum of nitrogen and sulfur content of sludge with respect to chlorine content of PVC e-waste, were studied. A total of 3 inhibition ratios were studied (0.25, 0.50, and 0.75), following the evidence that sulfur and nitrogen compounds inhibit the formation of pollutants such as PCDD/Fs in combustion processes.

In addition to the influence of inhibition ratio, the effect of the levels of oxygen in the combustion atmosphere was investigated too by introducing the concept of lambda (λ), which refers to the amount of oxygen available to react with the sample during its combustion. The objective was to evaluate how oxygen affected the formation of these pollutants. Two different oxygen ratios (λ = 0.15 and λ = 0.50) were considered, searching to recreate incomplete combustion conditions that can occur in these situations.

The results of analyzed emissions were divided into 4 families of compounds: The first dedicated to the study of PAHs, the second to ClBzs and ClPhs, the third to PCDD/Fs and, finally, the fourth, dedicated to dl-PCBs. In order to select the best operating conditions for reducing the whole pollutant emissions, the main conclusions obtained in each block are summarized here:

- Regarding PAHs, when working in poor oxygen conditions (λ = 0.15) the best results were obtained for an inhibition ratio of 0.75, reducing some of the compounds by up to 60% with respect to the expected theoretical formation. However, the best results were obtained for λ = 0.50, where there was a significant reduction in the formation of pollutants for all inhibition ratios, being 0.75 the best inhibition ratio.
- The concentrations obtained for ClBzs and ClPhs were in many cases near zero, especially for ClPhs (except for 3,5-dichlorophenol). For these compounds, not all the factors mentioned above were analyzed due to the few values that could be obtained (most of the results being under the detection limit). However, it was enough to find a clear and visible trend. It was determined that for ClBzs in poor oxygen conditions (λ = 0.15), the best inhibition ratio was 0.50, while for higher oxygen ratios (λ = 0.50), the best inhibition ratio was 0.75. As for PAHs, the lowest ClBzs emissions were obtained when working at λ = 0.50. For ClPhs, the results obtained were similar to those obtained for ClBzs, but in this case for both oxygen ratios the best inhibition ratio was 0.75.
- Regarding the analysis of PCDD/F emissions, the best inhibition ratio was 0.75 for both oxygen ratio conditions. However, for poor oxygen conditions (λ = 0.15), all inhibition ratios caused an increase in the formation of PCDD/Fs with respect to the expected theoretical amount. On the other hand, for a higher oxygen ratio (λ = 0.50), a very significant reduction in the emission of all PCDD/Fs was observed for every mixture, being 0.75 the best inhibition ratio. In addition, the global inhibition efficiencies reached were 89.2%, 71.4%, and 98.8% for the inhibition ratios 0.25, 0.50, and 0.75, respectively.
- Finally, the results regarding dl-PCB emissions followed a similar trend to what was detected for all the previous analyzed compounds. When the emissions were produced with a λ = 0.15, the best inhibition ratio was 0.50. However, an increase in the formation of PCBs was observed for all inhibition ratios, with respect to the expected theoretical values. PCB emissions in a higher oxygen presence (λ = 0.50) offered the best results for an inhibition ratio of 0.75, too. In addition, there was a significant reduction of emissions for every inhibition ratio, between 60% and 95% of inhibition. Finally, the global inhibition efficiencies achieved were 86.7%, 76.5%, and 91.9% for the inhibition ratios 0.25, 0.50, and 0.75, respectively.

To conclude, the optimal conditions for a general inhibition effect in every pollutant studied were achieved under higher oxidative conditions (λ = 0.50). Moreover, the addition of sludge to PVC e-waste combustion was really convenient, working with inhibition ratios R_i_ ≥ 0.25, being 0.75 the inhibition ratio that delivered the best results in most cases.

Thus, it would be interesting to study these emissions on further investigations for a molar inhibition ratio of 0.8, which correspond exactly to a mass ratio 1:1 (in this case, with this particular PVC e-waste and sludge compositions), being able to simplify as much as possible the mixing process during its co-incineration and being beneficial for its possible future industrialization and inhibition capacity improvement.

The results also revealed a great sensitivity to the amount of oxygen available during the reaction in sub-stochiometric conditions, so it will be crucial to have this parameter well controlled, especially in incinerators, where this element can be scarce in some points and incomplete reactions are frequent.

## Figures and Tables

**Table 1:** Characterization of PVC e-waste in a proportion of 82.8 wt% of PVC and 17.2 wt% of PE [28].

Elemental Analysis	wt%
C	43.16
H	6.20
N	*nd*
**S**	*nd*
Cl	22.57
Ca	19.46
Si	0.07
O	8.15

nd: Not detected.

**Table 2:** Characterization of sewage sludge (wt%).

**Elemental Analysis**	**wt%**
C	42.70
H	6.05
N	6.77
S	1.18
O by difference	22.74
Ash content	20.56
**X-ray Fluorescence Analysis**	**wt%**
Na	0.34
Mg	0.88
Al	0.54
Si	1.33
P	3.48
Cl	0.65 *
K	0.61
Ca	6.73
Ti	0.27
Cr	0.01
Mn	0.02
Fe	5.14
Ni	0.01
Cu	0.08
Zn	0.17
Br	0.03 *
Sr	0.17
I	0.02
Ba	0.03
W	0.03
Pb	0.02

* Chlorine and bromine were also determined by ion chromatography using the methods 5050 and 9056A, with the results mentioned in the main text.

**Table 3:** List of experimental operating conditions.

SAMPLE ID	Load	λ	R_i_
1	Blank	-	-
2	Sludge	0.15	-
3	PVC e-waste	0.15	-
4	Sludge + PVC e-waste	0.15	0.25
5	Sludge + PVC e-waste	0.15	0.50
6	Sludge + PVC e-waste	0.15	0.75
7	Sludge	0.50	-
8	PVC e-waste	0.50	-
9	Sludge + PVC e-waste	0.50	0.25
10	Sludge + PVC e-waste	0.50	0.50
11	Sludge + PVC e-waste	0.50	0.75

## Data Availability

The datasets generated and analysed in the current study are available upon reasonable request to the corresponding author.

## Supplementary Materials

The following are available online at https://www.mdpi.com/2073-4360/13/5/835/s1. Four pages of additional information including Tables S1–S3, were available in the Supplementary Materials.
