# Supplementary material for: A Win–Win Combination to Inhibit Persistent Organic Pollutant Formation via the Co-Incineration of Polyvinyl Chloride E-Waste and Sewage Sludge
Source: Polymers (Basel). 2021 Mar 9;13(5):835. doi: 10.3390/polym13050835 (PMC7967143; doi:10.3390/polym13050835)
Supplement: Supplementary file 1 [file polymers-13-00835-s001.pdf]

polymers

MDPI

Supplementary Material

A win-win combination to inhibit persistent organic pollutant formation via the co-incineration of polyvinyl chloride e-waste and sewage sludge

Gerard Gandon-Ros [1,2*], Samuel S. Nuñez [1,2], Nuria Ortuño [1], Ignacio Aracil [1,2], María Francisca Gómez-Rico [1,2] and Juan A. Conesa [1,2]

[1] Institute of Chemical Process Engineering, University of Alicante, P.O. Box 99, E-03080 Alicante (Spain)

[2] Department of Chemical Engineering, University of Alicante, P.O. Box 99, E-03080 Alicante (Spain)

* Correspondence: ja.conesa@ua.es

The SM contains 4 pages of additional information and includes three tables.

Table S1. Instrument conditions for the analysis of PCDD/Fs and dioxin-like PCBs by GC-MSMS.

**GC conditions**	
Column	Agilent DB-5 MS UI (60 m × 250 µm × 0.25 µm)
Injection volume	1 µL
Injector temperature	300 ºC
Injection mode	Splitless
Oven program for PCDD/Fs	Initial: 80 °C (2 minutes)<br>Ramp at 60 °C/min to 140 °C (no hold)<br>Ramp at 20 °C/min to 200 °C (1 minute)<br>Ramp at 3 °C/min to 300 °C (7 minutes)
Oven program for dl-PCBs	Initial: 90 °C (1 minute)<br>Ramp at 20 °C/min to 180 °C (1 minute)<br>Ramp at 3 °C/min to 285 °C (no hold)
He (carrier gas) flow	1 mL/min
**MS conditions**	
Operation mode	Electron ionization (EI)<br>Multiple Reaction Monitoring (MRM)
Ionization voltage	70 eV
Transfer line temperature	290 ºC
Source temperature	280 ºC
Quadrupole temperature	150 ºC
MS resolution	Unit
He (quench gas) flow	2.25 mL/min
N_2 (collision gas) flow	1.5 mL/min

Table S2. MSMS parameters and collision energy for the analysis of native and ^{13}C-labelled PCDD/Fs.

Time segment	Analyte	Precursor ion (m/z)	Product ion (m/z)	Dwell (ms)	Collision energy (eV)
1 TCDD/F	TCDF	303.9	240.9	100	40
		305.9	242.9	100	40
	^{13}C-TCDF	315.9	251.9	20	40
		317.9	253.9	20	40
	TCDD	319.9	256.9	100	26
		321.9	258.9	100	26
	^{37}Cl-TCDD	327.9	198	20	40
		327.9	263	20	26
	^{13}C-TCDD	331.9	267.9	20	26
		333.9	269.9	20	26
2 PeCDD/F	PeCDF	337.9	274.9	100	40
		339.9	276.9	100	40
	^{13}C-PeCDF	349.9	285.9	20	40
		351.9	287.9	20	40
	PCDD	353.9	290.9	100	26
		355.9	292.9	100	26
	^{13}C-PeCDD	365.9	301.9	20	26
		367.9	303.9	20	26
3 HxCDD/F	HxCDF	373.8	310.9	100	40
		375.8	312.9	100	40
	^{13}C-HxCDF	385.8	321.9	20	40
		387.8	323.9	20	40
	HxCDD	389.8	326.9	100	26
		391.8	328.8	100	25
	^{13}C-HxCDD	401.8	337.9	20	26
		403.8	339.8	20	26
4 HpCDD/F	HpCDF	407.8	344.8	100	40
		409.8	346.8	100	40
	^{13}C-HpCDF	419.8	355.8	20	40
		421.8	357.8	20	40
	HpCDD	423.8	360.8	100	24
		425.8	362.8	100	24
	^{13}C-HpCDD	435.8	371.8	20	26
		437.8	373.8	20	24
5 OCDD/F	OCDF	441.7	378.8	150	40
		443.7	380.8	150	40
	OCDD	457.7	394.8	150	24
		459.7	396.8	150	24
	^{13}C-OCDD	469.7	405.8	20	24
		471.7	407.8	20	24

Table S3. MSMS parameters and collision energy for the analysis of native and ^{13}C-labelled dioxin-like PCBs.

Time segment	Analyte	Precursor ion (m/z)	Product ion (m/z)	Dwell (ms)	Collision energy (eV)
1 tetra- and penta-PCBs	tetra-PCB	289.9	219.9	200	25
		291.9	221.9	200	25
	^{13}C-tetra-PCB	301.9	232	20	25
		303.9	234	20	25
	^{13}C-penta-PCB	335.9	265.9	20	30
		337.9	267.9	20	30
2 penta- and hexa-PCBs	penta-PCB	323.9	253.9	200	30
		325.9	255.9	200	30
	^{13}C-penta-PCB	335.9	265.9	20	30
		337.9	267.9	20	30
	^{13}C-hexa-PCB	369.9	299.9	20	30
		371.9	301.9	20	30
3 hexa- and hepta-PCBs	hexa-PCB	357.8	287.9	100	30
		359.8	289.9	100	30
	^{13}C-hexa-PCB	369.9	299.9	20	30
		371.9	301.9	20	30
	hepta-PCB	393.8	323.9	100	30
		395.8	325.9	100	30
	^{13}C-hepta-PCB	405.8	335.8	20	30
		407.8	337.8	20	30

L: labelled
